# Ectopic Expression of OsPYL/RCAR7, an ABA Receptor Having Low Signaling Activity, Improves Drought Tolerance without Growth Defects in Rice

**DOI:** 10.3390/ijms21114163

**Published:** 2020-06-11

**Authors:** Nikita Bhatnagar, Rigyeong Kim, Seungsu Han, Jaeeun Song, Gang Seob Lee, Sangho Lee, Myung Ki Min, Beom-Gi Kim

**Affiliations:** 1 Metabolic Engineering Division, National Institute of Agricultural Sciences, RDA, Jeonju, Jeollabuk-do 54874, Korea; nikita.bhatnagar4@gmail.com (N.B.); jin4021766@gmail.com (R.K.); icanje@korea.kr (J.S.); 2 Department of Biological Sciences, Sungkyunkwan University, Suwon 16419, Korea; hsspoket@daum.net (S.H.); sangholee@skku.edu (S.L.); 3 Biosafety Division, National Institute of Agricultural Sciences, RDA, Jeonju, Jeollabuk-do 54874, Korea; kangslee@korea.kr

**Keywords:** abiotic stress signaling, ABA signaling, crop, growth and stress tolerance balance

## Abstract

Overexpression of abscisic acid (ABA) receptors has been reported to enhance drought tolerance, but also to cause stunted growth and decreased crop yield. Here, we constructed transgenic rice for all monomeric ABA receptors and observed that only transgenic rice over-expressing *OsPYL/RCAR7* showed similar phenotype with wild type, without total yield loss when grown under normal growth condition in a paddy field. Even though transgenic rice over-expressing *OsPYL*/*RCAR7* showed neither an ABA-sensitivity nor an osmotic stress tolerance in plate assay, it showed drought tolerance. We investigated the ABA-dependent interaction with OsPP2CAs and ABA signaling induction by OsPYL/RCAR7. In yeast two hybrid assay, OsPYL/RCAR7 required critically higher ABA concentrations to interact with OsPP2CAs than other ABA receptors, and co-immunoprecipitation assay showed strong interaction under ABA treatment. When ABA-responsive signaling activity was monitored using a transient expression system in rice protoplasts, OsPYL/RCAR7 had the lowest ABA-responsive signaling activity as compared with other ABA receptors. OsPYL/RCAR7 also showed weak suppression of phosphatase activity as compared with other ABA receptors in vitro. Transcriptome analysis of transgenic rice over-expressing *OsPYL/RCAR7* suggested that only a few genes were induced similar to control under without exogenous ABA, but a large number of genes was induced under ABA treatment compared with control. We conclude that OsPYL/RCAR7 is a novel functional ABA receptor that has low ABA signaling activity and exhibits high ABA dependence. These results lay the foundation for a new strategy to improve drought stress tolerance without compromising crop growth.

## 1. Introduction

Rice is a staple crop that provides a major source of calories for more than 2 billion people around the world, particularly in Asian countries. In the face of climate change and an increasing population, enhancing rice productivity is critical [1,2,3]. Thus far, several different kinds of drought tolerance regulatory genes, such as rice dehydration responsive element binding protein (OsDREB), basic leucine-zipper (OsbZIP), and ABA receptors, have been reported in various plant species [4,5,6,7,8]. Although ectopic expression of these genes strongly enhances drought tolerance, their overexpression causes severe defects in plant growth and seed yield [6,9,10]. Thus, stress-inducible promoters or co-expression of stress-tolerance and growth-regulatory genes are used to overcome these disadvantages [9,11,12]. However, maintaining the balance between crop productivity and stress tolerance under both normal and stress conditions is a major challenge encountered by crop scientists.

Abscisic acid (ABA) is a key stress hormone that regulates responses to osmotic stress, cold, and drought by negatively regulating overall development [13,14,15]. The biosynthesis of ABA starts in plastids and completes in cytosol. Furthermore, inactive ABA conjugated with glucose is converted to an active state in immediate stress response [13,15,16]. A sudden increase in ABA concentration acts as a signal under different abiotic stress conditions. Under osmotic stress, the inactive ABA glucose ester (ABA-GE) is rapidly dissociated by β-glucosidase, forming active ABA that relocates to the endoplasmic reticulum [13]. Intracellular perception of ABA is initiated by both an ABA receptor PYRABACTINE RESISTANCE 1/PYR1-LIKE/REGULATORY COMPONENTS OF ABA RECEPTOR (PYR/PYL/RCAR) and a Clade A Type 2C phosphatase (PP2CA), also considered as co-receptors [16,17,18,19]. The presence of ABA induces the formation of triple complex (ABA receptor-ABA-PP2CA), which activates the SNF1-related kinases 2 (SnRK2s). The activated SnRK2s initiates ABA signaling by activating downstream bZIP transcription factors and activating anion channels such as slow anion channel-associate 1 (SLAC1) [19,20,21,22].

Several ABA receptors have been functionally identified in both monocot and dicot lineages of plant kingdom. A total of 14 ABA receptors have been reported in Arabidopsis and 13 in rice [6,17,23,24,25,26,27,28,29,30,31,32,33,34]. Higher order mutants in Arabidopsis show high ABA insensitivity [19]. The analysis of higher order mutants in rice was reported recently with combinatorial mutants of class I and class II ABA receptors [35]. Distinct mutant phenotype with higher growth rate was shown for class I mutation and no significant phenotype was recorded for class II mutants. These differences among the ABA receptors suggest the possibility of their functional variation between different classes. Apart from the mutant analyses, earlier studies have shown that gain-of-function mutations of ABA receptors negatively regulate plant growth and positively enhance the osmotic stress tolerance and sensitivity to ABA [6,25,28,34,36]. On the contrary, few ABA receptors have been reported to be involved in other signaling pathways as well. *AtPYL4* is involved in jasmonate signaling, and *AtPYL9* promotes leaf senescence [35,36]. The regulation of water use efficiency (WUE) in wheat by alteration in the ABA receptors provides another scenario of fine tuning the ABA receptors expression in plants and developing osmotic stress tolerant crops without compromising the total yield [37,38].

ABA receptors exist in monomeric and dimeric forms. The dimeric forms require ABA to dissociate into monomers and form complexes with downstream targets PP2CAs, whereas monomeric ABA receptors may or may not require ABA to interact with PP2CAs [25,28,39,40]. On the basis of different ABA concentrations, the intensity of interaction of monomeric ABA receptors with PP2CAs might determine the specific cellular functions of ABA [41]. RCAR1 and RCAR3 receptors, which are very similar in terms of amino acid sequence, differ in sensitivities for ABA and suppressive activities for ABI1 and ABI2 [30]. However, to date, the biological relevance of each ABA receptor’s different affinity for ABA under stress conditions has not been well studied.

Here, we show that the functional monomeric ABA receptor OsPYL/RCAR7 has the lowest ABA-mediated interaction affinity and ability to suppress the activity of OsPP2CAs among all monomeric ABA receptors investigated. With inherent lower ABA sensitivity, the overexpression of *OsPYL/RCAR7* resulted in no growth retardation and yield penalty or major morphological changes under normal growth conditions. However, under drought stress condition, plants showed a higher survival rate compared with the wild type (WT) in young seedling stages. Thus, OsPYL/RCAR7 may be a unique ABA receptor that can be used to enhance drought tolerance without inducing any growth defects in crops. This work provides a new strategy to develop abiotic stress-tolerant crops without yield penalty by introducing stress tolerance mechanisms that are not activated under normal or mild stress conditions, but function specifically under harsh stress condition.

## 2. Results

### 2.1. Ectopic Expression of OsPYL/RCAR7 Inhibits Neither Growth nor Seed Yield in Normal Growth Conditions

We reported previously that transgenic rice lines over-expressing *OsPYL5* showed a strong drought stress tolerance and hypersensitivity for ABA with severe growth retardation and reduced seed yield in paddy field [6]. In addition, according to Miao and co-worker’s report (2018), the knock-out lines of ABA receptors showed an increase in growth under paddy field conditions, suggesting that each ABA receptor affects plant growth differently. Overexpression of *TaPYL4* showed the increase of ABA sensitivity, resulting in higher WUE and drought tolerance in wheat [38]. To select the ABA receptors exhibiting optimal growth and lack of total yield penalty, we generated overexpression lines of seven rice monomeric ABA receptors and observed the growth phenotype in paddy field. Except for *OsPYL/RCAR7*, all other overexpression plants of ABA receptors showed growth retardation and lower crop yield, as reported earlier [6] (Figure S1).

**Figure 1:**
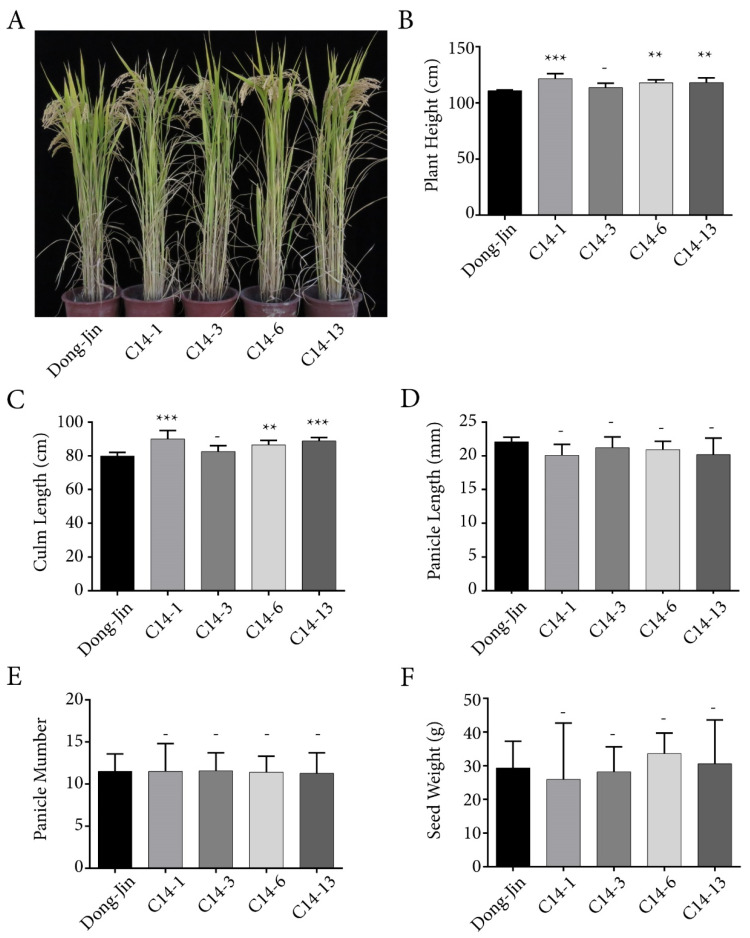
Agricultural traits of rice over-expressing *OsPYL*/*RCAR7* in paddy field. (**A**) Rice plants grown in a paddy field. Image was recorded just before the harvest. (**B**–**F**) Graphical representation of quantified data of the major agricultural traits of rice productivity. Values represent the average and error bars depict standard deviation. Data were recorded from at least 20 individual plants per line. ** means *p*-value is less than 0.01. *** means *p*-value is less than 0.001. – means *p*-value is higher than 0.05.

For detailed phenotype analysis of plants over-expressing *OsPYL/RCAR7* (named as C14s), four independent lines of C14s were chosen. The agricultural traits were examined in comparison with the transformation background rice cultivar Dongjin in paddy field. The over-expression of C14s was confirmed by quantitative reverse transcription polymerase chain reaction (RT-qPCR) analysis (Figure S2). Three of them showed a slight increase in plant height and culm length and one was similar to Dongjin plants (Figure 1A–C). At yield analysis, the over-expression lines of *OsPYL*/*RCAR7* showed panicle length, panicle number, and seed weight similar to that of Dongjin plant (Figure 1D–F). There were no significant differences in agricultural traits between the gain-of-function transgenic plants and wild-type Dongjin plants. These investigations were repeated annually and their results were nearly identical for three years. These results suggest that, unlike other ABA receptors, overexpression of *OsPYL/RCAR7* shows no defect in plant growth under normal paddy field conditions.

### 2.2. Ectopic Expression of OsPYL/RCAR7 Showed No Responses to ABA and Osmotic Stress, but Confers Drought Tolerance in Vegetative Stage

**Figure 2:**
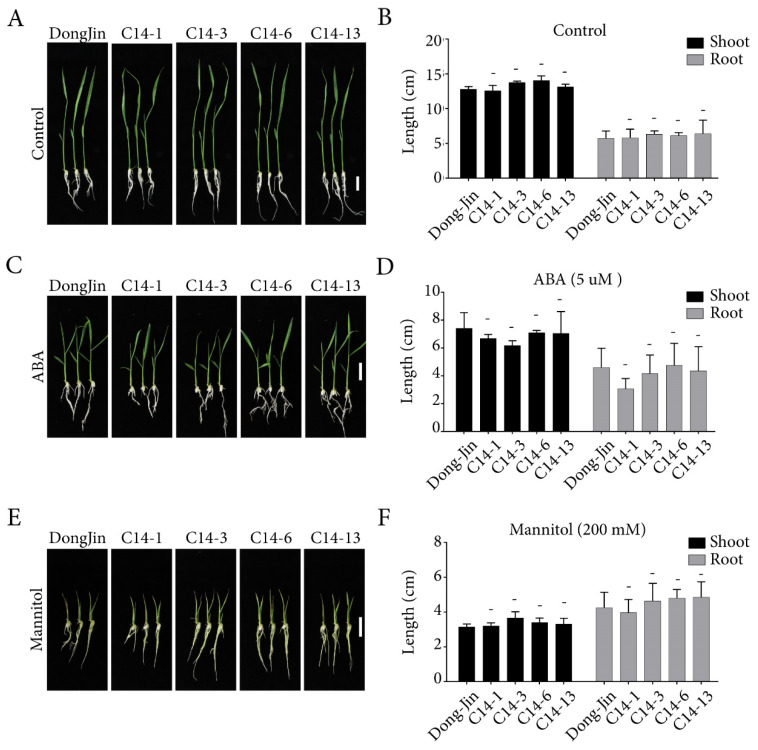
Post-germination assay of transgenic rice lines overexpressing *OsPYL*/*RCAR7* under abscisic acid (ABA) treatment and osmotic stress conditions. (**A**,**C**,**E**) Representative images of rice seedlings grown on media, 1/2MS, supplemented with 5 μM ABA and 200 mM mannitol. (**B**,**D**,**F**) Quantitiative data of the post-germination assay. No significant differences were detected. The graphs show average values with standard error (*n* = 5). Three indepent experiments were performed and showed similar results in every repeats. Representative results were presented. – means no significance.

**Figure 3:**
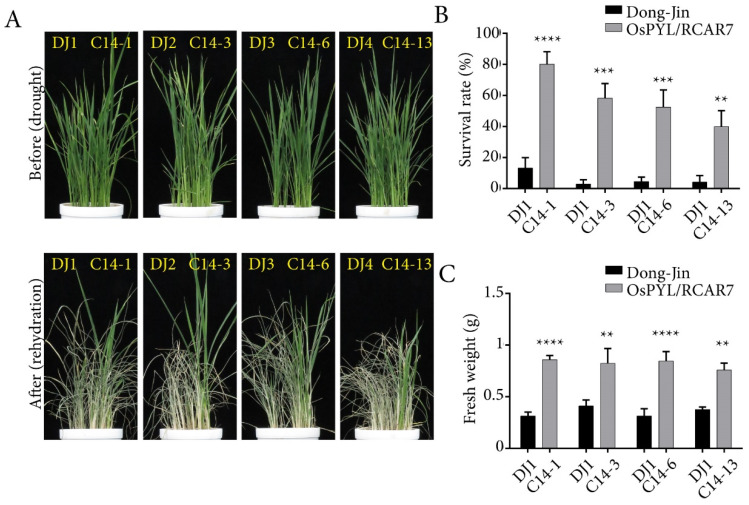
Transgenic rice over-expressing *OsPYL*/*RCAR7* showed drought tolerance in vegetative stage. (**A**) Representative images of plants grown in the drought tolerance assay. All plants over-expressing *OsPYL/RCAR7* (C14s) had the higher survival rate than wild type (WT) (DJs). Upper panel represents plants before drought stress treatment. Lower panel represents plants after drought stress treatment. (**B**) Graphical representation of survival rate after drought stress. Survival rate was calculated by counting the percentage of surviving plants in each pot. (**C**) Graphical representation of quantified fresh weight after drought stress. The graphs show average values with standard error (*n* = 6). The assay was conducted in triplicate and showed similar results. Plants were subjected to drought treatment for 7–10 days before recommencing watering. ** means *p*-value is less than 0.01,*** means *p*-value is less than 0.001 and **** means *p*-value is less than 0.0001.

Overexpression of ABA receptors in rice has been reported to show extreme growth inhibition under the presence of exogenous ABA [6,25,35]. To characterize the effects of *OsPYL/RCAR7* over-expression in ABA and stress responses, we performed the post germination assay using four C14 transgenic lines in normal condition, presence of ABA, and in osmotic stress condition (Figure 2). Contrary to reported phenotype of ABA receptor over-expressing plants, the C14 transgenic lines showed no response under normal or stress condition as compared with WT. We quantified the length of shoots and roots and observed no significant difference between WT and C14 plants (Figure 2). We examined the C14 plants for drought tolerance in soiled pots in green house. Similar to the plate assays, the C14 transgenic lines under normal condition showed a growth pattern like that of WT. The rehydrated transgenic plants in the soiled pots were observed to be greener and less wilted as compared with the WT (Figure 3A). On quantification, the survival rate of transgenic lines was significantly higher by 40–80% as compared with the WT. Similarly, fresh weight was significantly increased in transgenic lines as compared with WT (Figure 3B,C). These results suggest that, though no stress or ABA response was observed in C14 plants in the controlled conditions on plates, the gain-of-function mutants are able to rescue the plants under severe drought conditions in soil.

### 2.3. Comparative Transcriptome Analysis between Plants Over-Expressing OsPYL/RCAR7 or OsPYL/RCAR3

**Figure 4:**
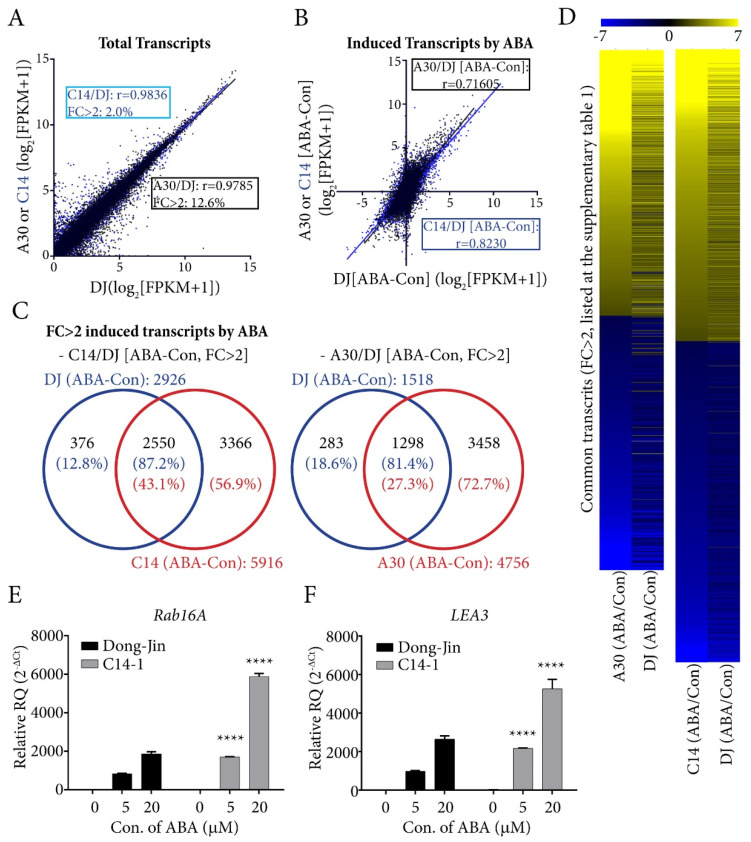
Comparative transcriptome analysis of transgenic rice overexpresing *OsPYL/RCAR7* and *OsPYL/RCAR3*. (**A**) Total transcripts of C14 (transgenic plants over-expressing *OsPYL/RCAR7*) (blue) and A30 (transgenic plants over-expressing *OsPYL/RCAR3*) (black) treated with no ABA were compared with DJ (wild type). ‘*r*’ is pearson correlation coefficient. FC is fold change. (**B**) Induced or reduced transcripts in C14 (blue) or A30 (black) treated with ABA were compared. (**C**) Venn diagram of transcripts with more than twofold changes under ABA treatment. (**D**) Heat map of common trascripts (fc > 2). (**E**,**F**) Quantitative reverse transcription polymerase chain reaction (RT-qPCR) analysis with ABA dependently induced gene. Each transcript was detected in treated samples with indicated ABA treatment for 12 h. Similar expression levels were observed in triplicates. Error bars are SD. **** means that *p*-value is less than 0.0001. FPKM, fragment per kilo base of transcript per million mapped reads.

Plants over-expressing *OsPYL/RCAR7* showed no growth retardation and no hypersensitivity to ABA, but showed drought tolerance contrary to other plants over-expressing ABA receptors. To identify differences in terms of gene expression level, we carried out comparative analysis. The transcriptomes of rice over-expressing *OsPYL/RCAR7* (named as C14) and *OsPYL/RCAR3* (named as A30) were analyzed. *OsPYL/RCAR3* overexpression showed growth retardation and ABA hypersensitivity. We conducted the transcriptome analysis of C14 and A30 plants using 2-week-old seedlings grown under ABA treatment and normal growth condition. All aligned reads of transcripts were normalized by transforming to FPKM (fragment per kilo base of transcript per million mapped reads) and then to log_2_ fold change before comparison. When we compared the transcripts of C14 versus DJ and A30 versus DJ expressed at normal growth condition, both correlation coefficients (Pearson correlation coefficient: *r*) were significantly high (C14 vs. DJ: *r* = 0.9836, A30 vs. DJ: *r* = 0.9785). However, on comparison of total transcript change of more than twofold change as compared to WT, A30 comprises about 12.6%, whereas C14 accounted for only 2%. This means that C14 plants have almost similar transcriptome with wild type, but A30 plant has more induced or repressed transcriptome compared with wild type under normal growth condition (Figure 4A). Next, we compared the total transcripts of DJ, C14, or A30 changed by ABA treatment. The correlation coefficients of changed transcripts of ABA treated DJ, C14, or A30 were examined. The changed patterns of C14 transcriptome were closer to that of DJ (*r* = 0.8230) than those of A30 (*r* = 0.7160) (Figure 4B). Among the transcripts that were changed more than twofold by ABA treatment, 43.1% of C14 transcripts covered the 87.2% of DJ transcripts and 27.3% of A30 transcripts covered 81.4% of DJ transcripts (Figure 4C; Data S1). It was clear that transcripts were changed more in A30 than C14, and the transcriptomes of C14 plant were almost similar to WT in normal growth condition. However, transcriptomes of C14 were induced or repressed more than WT in ABA treatment condition. We confirmed these results by RT-qPCR analysis of ABA dependently induced genes (*Rab16A* and *LEA3*) in WT and C14 (Figure 4E). The transcriptome data provided evidence that the genes expressed in the overexpression lines of *OsPYL/RCAR7* showed a similar trend as that of WT under normal conditions, but responded more sensitively to ABA than WT.

### 2.4. OsPYL/RCAR7 Is a Functional ABA Receptor Featuring Stringent ABA Dependency and Low Signaling Effects

**Figure 5:**
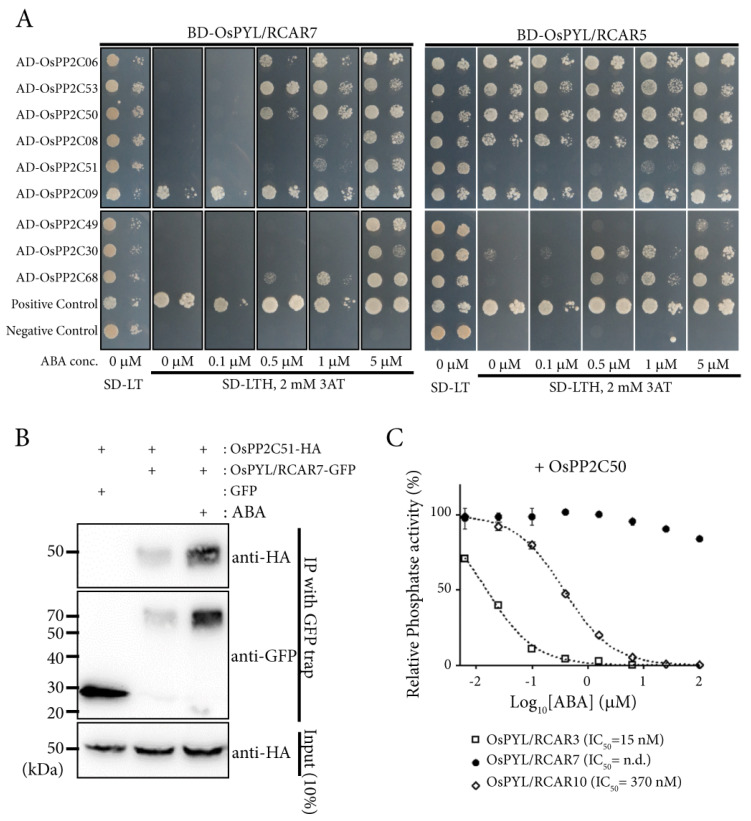
OsPYL/RCAR7 has stringent ABA dependency to interact with OsPP2CAs. (**A**) Yeast two hybrid analysis using OsPYL/RCAR7 and OsPYL/RCAR5 as baits and OsPP2CAs as preys. Synthetic minimal medium lacking leucine, tryptophan, and histidine (SD-LTH) supplemented with 0 to 5 μM concentration of ABA were used. Photographs were taken 5 days after inoculation of transformed yeasts. (**B**) OsPYL/RCAR7-GFP (green fluorescent protein) was immunoprecipiated and co-immunoprecipiated OsPP2C51 was detected by human influenza hemagglutinin (HA) antibody. (**C**) Inhibition assay of phosphatase activity for OsPPC50 was performed by three ABA receptors (OsPYL/RCAR3, 7, and 10).

**Figure 6:**
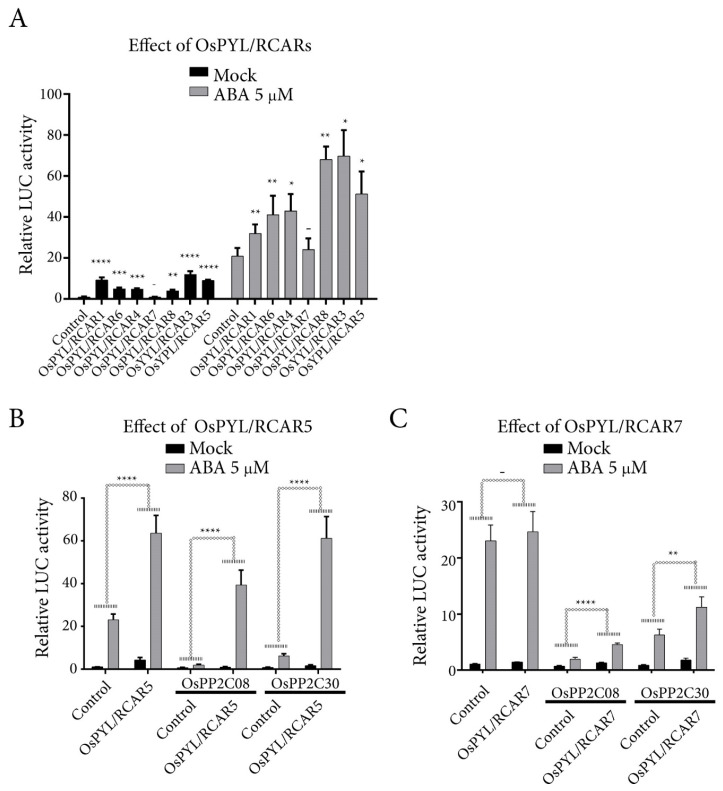
OsPYL/RCAR7 showed the lowest activity among monomeric ABA receptors. (**A**) Comparative analysis of ABA signaling effects of monomeric ABA receptors under absence or presence of ABA. Luciferase (LUC) assay was done using rice protoplasts, reporter, and ABA receptors as signaling effectors. (**B**,**C**) Comparative analysis of OsPYL/RCAR7 and OsPYL/RCAR5 activity to suppress individual phosphatase PP2C08 and OsPP2C30. Luciferase assay was done using rice protoplasts, reporter, OsPP2CAs, and ABA receptors. Luciferase activity was measured by comparing with co-transformed marker (Ubi::rLUC), fLUC/rLUC. Values represent averages and error bars are SD (*n* = 3). These experiments were repeated three times. Results showed similar patterns and representative results were presented. – means that *p*-value is more than 0.05, * means that *p*-value is less than 0.05, ** means that *p*-value is less than 0.01 and **** means that *p*-value is less than 0.0001.

To characterize molecular properties of *OsPYL/RCAR7*, we examined the interactions between clade A OsPP2Cs (OsPP2CAs), which are well known co-receptors for ABA, and two different ABA receptors OsPYL/RCAR7 and OsPYL/RCAR5 using yeast two hybrid. A monomeric ABA receptor OsPYL/RCAR5 showed the ABA-independent interactions with five OsPP2CAs and ABA-dependent interaction with four OsPP2CAs, whereas OsPYL/RCAR7 showed ABA independent interaction with only OsPP2C9. Moreover, OsPYL/RCAR7 required higher concentrations of ABA than OsPYL/RCAR5 for interaction with OsPP2C30 and OsPP2C68. Taken together, OsPYL/RCAR7 shows higher ABA-dependent interaction activity as compared with OsPYL/RCAR5 for most of the protein phosphatases (Figure 5A). In addition, we conducted the co-immunoprecipitation assay. The interaction strength of OsPYL/RCAR7 and OsPP2C51 increased in the presence of ABA (Figure 5B). In vitro phosphatase activity assay showed that the inhibition of OsPP2C50 using OsPYL/RCAR7 was significantly lower as compared with using the other ABA receptors OsPYL/RCAR10 or OsPYL/RCAR3 (Figure 5C). Next, we monitored how much each ABA receptor affects ABA signaling using the protoplast system in planta. Rice protoplasts were co-transformed with the reporter, an ABA responsive synthetic promoter fused to the firefly luciferase gene, and vector constructs to overexpress different monomeric ABA receptors [21,42]. Under control condition, OsPYL/RCAR1 and OsPYL/RCAR3 exhibited high activity as compared with all other ABA receptors. In the presence of 5 μM ABA, OsPYL/RCAR3 showed the highest ABA response, followed by OsPYL/RCAR8, 5, 4, 6, 1, and 7. Notably, this analysis showed that the response of OsPYL/RCAR7 was consistently low throughout under control as well as ABA treatment (Figure 6A). To check the inhibition activity of OsPYL/RCAR7 for each OsPP2Cs, which suppress the ABA signal transduction, we introduced the ABA receptors and OsPP2Cs into rice protoplasts for luciferase induction assay. In comparison with reported OsPYL/RCAR5 as a functional ABA receptor [6], OsPYL/RCAR7 was tested for its inhibition activity for OsPP2C08 and OsPP2C30, which showed higher ABA-dependent interaction in yeast two hybrid assay. In the case of OsPYL/RCAR5, both OsPP2C08 and OsPP2C30 showed suppressing activity under control condition, but clearly repressed the activity in the presence of ABA. In the presence of ABA, the responses were increased about 20-fold or 9-fold for the OsPP2C08 or OsPP2C30, respectively (Figure 6B,C). Contrary to OsPYL/RCAR5, OsPYL/RCAR7 showed that ABA signaling was increased by 134% or 79% in the presence of ABA for OsPP2C08 or OsPP2C30, respectively. Inhibition activity of OsPYL/RCAR7 for both OsPP2Cs was clearly shown in this experiment to be extremely low as compared with that of OsPYL/RCAR5. Taken together, OsPYL/RCAR7 is a novel intrinsic ABA receptor to interact with OsPP2Cs critically in high ABA concentrations and have low ABA signaling effects.

## 3. Discussion

Several cytosolic ABA receptors, PYL/RCARs, have been functionally identified to confer drought tolerance. Along with the stress tolerance phenotype, the overexpression of OsPYL/RCARs also causes ABA hypersensitivity, leading to plant growth inhibition. These phenotypes are quite similar in Arabidopsis, rice, and other plants. Plant growth inhibition is one of the bottle necks in the development of abiotic stress tolerant crops using ABA receptors [6,25,35,43,44]. Thus, it is important to identify the ABA receptors or characterize the mechanisms to show drought tolerance without growth defects. We suggest the possibility and concepts for the development of drought-tolerant crops without growth defects using abiotic stress signaling genes.

### 3.1. The Diverse Responses of ABA Receptors to ABA May Cause Biologically Different Stress Responses in Plants

Plants synthesize ABA in response to abiotic stresses such as drought, cold, and high salinity. The regulation of cellular ABA concentration depends on the intensity and duration of stress [45]. ABA receptors form complexes with PP2CAs, which, in vitro, have different ABA-binding activities and different interaction affinity with PP2CAs in Arabidopsis and rice [34,46]. The different ABA concentration-mediated interactions between ABA receptors and PP2Cs have also been previously reported in vivo for several plants [20,25,34]. These different sensitivities of ABA receptors to ABA concentrations might be important for regulating responses to diverse abiotic stress conditions. However, there is little direct evidence for this because of functional redundancy. Single knockout mutants of ABA receptors did not show a phenotype and tri- or quadruple mutants only showed insensitive phenotypes to the ABA [34]. Thus, it is difficult to elucidate differential phenotypes in terms of ABA sensitivity among PYL/RCARs.

In this study, we used gain-of-function approach to generate over-expressing OsPYL/RCARs transiently and permanently. Both resulted in monitoring the ABA signaling output to compare the ABA responsiveness of each OsPYL/RCAR. OsPYL/RCAR7 had the lowest ABA sensitivity in phenotypes and signaling effects among ABA receptors, indicating that it is a good model to investigate the possible function of ABA receptors having low sensitivity to ABA in planta. Over-expression transgenic lines of *OsPYL/RCAR7* showed no growth retardation and reduced yield under normal growth condition, but showed drought tolerance. These unique phenotypes of plants over-expressing *OsPYL/RCAR7* can be explained at the molecular level by strict ABA dependency of *OsPYL/RCAR7*. Over-expression of *OsPYL/RCAR7* activated transcription of ABA-responsive/stress tolerant genes higher than WT only under ABA treatment, but not under normal growth condition. Moreover, OsPYL/RCAR7 did not show ABA-independent interaction with OsPP2CAs except for OsPP2C9 and required higher ABA concentrations than other ABA receptors. Taken together, *OsPYL/RCAR7* is not able to activate ABA signaling at low ABA concentrations or in mild stress and normal growth condition. However, it is able to function at high ABA concentrations or in harsh stress conditions such as drought stress. Thus, differing sensitivities of ABA receptors to ABA concentration might be important to balance growth/productivity and stress tolerance.

### 3.2. Regulatory Genes That Are Preferentially Functional under Severe Stress Conditions Might Be Good Candidates to Develop Abiotic Stress-Tolerant Crops without Growth Defects

Thus far, several abiotic stress-tolerance genes such as DREB, bZIP, SnRK2, and so on have been identified by several research groups and these genes are very sensitive to even mild stresses or low ABA concentration conditions. Thus, the ectopic expression of those genes in plants invokes stress responses under normal growth conditions, resulting in stunted growth and yield penalties. To overcome these problems, a stress-inducible promoter such as *RD29a* has been applied to these genes to induce their expression only under stress conditions. After the *RD29a* promoter-fused *AtDREB1* successfully increased drought stress tolerance without growth inhibition, this approach was successfully applied in other genes and several crops to develop the stress-tolerant crops [47].

Some stress-tolerance genes have been reported to increase tolerance without incurring growth inhibition and yield penalty even though molecular mechanisms are not investigated well [48]. In this study, the comparison between transgenic lines over-expressing *OsPYL/RCAR7* and other monomeric ABA receptors shows that the low sensitivity to ABA minimizes the growth defects even though it has low abiotic stress tolerance. However, a hypersensitive ABA receptor, OsPYL/RCAR5, showed growth defects under normal and abiotic stress conditions even though it has strong abiotic stress tolerance. We suggest a new strategy and molecular mechanism for the introduction of stress tolerance without incurring growth inhibition. These phenotypes explain that the over-expression of *OsPYL/RCAR7* accentuates the drought stress tolerance because drought stress can sufficiently increase the ABA concentration in cells where OsPYL/RCAR7 functions as an ABA receptor. Unlike other OsPYL/RCARs reported in previous studies, *OsPYL/RCAR7* gene has no influence on the overall growth of over-expression plants, resulting in maintenance of crop yield under normal field conditions because *OsPYL/RCAR7* gene might not function as an ABA receptor at low ABA concentrations and under mild stress and normal growth conditions. Thus, new approaches to develop genes that are differentially activated in stress conditions, and investigation of the regulation mechanisms under different stress intensities, might be fruitful to develop the stress-tolerant crops without incurring growth defects.

## 4. Materials and Methods

### 4.1. Generation of Transgenic Rice

The rice cultivar used in this study was *Oryza sativa* cv. Dongjin. The complete coding sequence (CDS) of *OsPYL/RCAR7* (LOC_Os06g33640) was cloned into the pGA2897 gateway vector containing the maize *ubiquitin* promoter to generate overexpression (OX) transgenic rice plants [30,42]. Transgenic rice plants were prepared by *Agrobacterium*-mediated transformation, as reported previously [34]. Transgenic plants were selected on half-strength Murashige and Skoog (½ MS) medium (DUCHEFA BIOCHEMIE B.V, Haarlem, Netherlands) supplemented with 40 μg/mL hygromycin (DUCHEFA BIOCHEMIE B.V, Haarlem, Netherlands). Quantitative reverse transcription polymerase chain reaction (RT-qPCR) analysis was conducted by isolating RNA from overexpression lines using the RNeasy Plant Mini Kit (Qiagen, Hilden, Germany).

### 4.2. Agricultural Trait Analysis

Transgenic plants were grown on ½ MS medium containing hygromycin B (40 mg/L, DUCHEFA BIOCHEMIE B.V, Haarlem, Netherlands) for selection. After incubating for 1 week under long day conditions (16 h light/8 h dark), seedlings were transferred to pots for acclimation in a greenhouse. After 4 weeks, seedlings were planted in paddy fields during the month of May, and phenotypes were observed at regular intervals until harvest in October, as described previously [6]. Plant height, panicle length, panicle number, and seed weight were recorded for approximately 20 plants per line. Data were summarized, and average values were calculated and assembled as graphs.

### 4.3. Post-Germination Assay

Sterilized rice seeds were plated on ½ MS medium supplemented with hygromycin for 3–4 days. Young seedlings were transferred onto ½ MS medium as the control, and ½ MS medium supplemented with 5 µM ABA (Sigma-Aldrich Corporation, St. Louis, MO, USA) or 200 mM mannitol (DUCHEFA BIOCHEMIE B.V, Haarlem, Netherlands). Plates were incubated for 5–7 days. Growth differences were recorded by measuring root and shoot length, and an average of the total measurements was calculated and presented as a graph.

### 4.4. Yeast Two Hybrid Assay

The Matchmaker GAL4 two hybrid system 3 (Clontech Laboratories, Takara Bio USA Inc., Mountain View, CA, USA) was used. Complete CDSs of *OsPP2C08*, *OsPP2C51* (Os05g49730), *OsPP2C30*, and *OsPP2C68* (Os09g15670) were cloned into the *pGADT7* vector, and CDSs of *OsPYL/RCAR1*, *OsPYL/RCAR5*, *OsPYL/RCAR6*, and *OsPYL/RCAR7* were cloned into the pGBKT7 vector. Constructs were arranged in several combinations and cotransformed into *Saccharomyces cerevisiae* strain AH109 (*MATa*, *trp1-901*, *leu2-3*, *112*, *ura3-52*, *his3200*, *gal4*Δ, *gal80*Δ, *LYS2::GAL1UAS-GAL1TATA-HIS3*, *GAL2UAS-GAL2TATA-ADE2*, *URA3::MEL1UAS-MEL1TATA-lacZ*, *MEL1*). Cells were plated on synthetic minimal medium (Clontech Laboratories, Takara Bio USA Inc. CA, USA) lacking leucine and tryptophan (SD-LT), and were subsequently streaked onto media lacking leucine, tryptophan, and histidine (SD-LTH), supplemented with 2 mM 3-AT (3-amino-1, 2, 4-triazole) and increasing concentrations of ABA. Plates were incubated at 30 °C for 3 days.

### 4.5. Luciferase Assay

Luciferase assays were conducted as described previously [30]. Complete CDSs of *OsPYL/RCAR7*, *OsPYL/RCAR5*, *OsPP2C08*, and *OsPP2C30* were cloned into the pGEM-UbiFLAG transient expression vector, resulting in protein fusion with FLAG-tag. Constructs were transformed into protoplast cells and incubated overnight (20 h) under control (without ABA) and 5 µM ABA treatments. Samples were harvested by removing excess W5 solution, and 50 µL of passive-lysis buffer (Promega Cooperation, Madison, WI, USA) was immediately added before freezing in liquid nitrogen. Samples were vortexed, and luciferase activity was detected using a dual luciferase assay system according to the manufacturer’s protocol (Promega Cooperation, Madison, WI, USA) for 10 µL of lysed cells. pAtUBQ-rLUC (Renilla luciferase) was used as an internal control and pRab16A-fLUC promoter was used to monitor ABA signaling activity.

### 4.6. Drought-Tolerance Assay

Drought-tolerance assays were performed as described previously [6]. Transgenic T3 seeds overexpressing *OsPYL*/*RCAR7* were sterilized and germinated in distilled water containing hygromycin (40 μg/mL) for 5–6 days. Selected seedlings were transferred to soil and kept in a greenhouse for 21 days. Plants were watered regularly, and then watering was ceased for 8–9 days to simulate drought conditions, until plants completely wilted, at which point they were re-watered. When phenotypes were clearly visible, data were recorded for all samples in each pot. Survival rate was measured by counting the percentage viable plants in each pot. Fresh weight was recorded by weighing the tissue on scale. Three replicate pots were used for each overexpression line and control combination.

### 4.7. RNA Sequencing Analysis

For the transcriptomic analysis, we prepared the total RNA from 14-day-old plant grown on ½ MS media. C14 (OsPYL/RCAR7 overexpressing plant) and its control Dongjin plants were prepared with or without ABA treatment for 24 h. A30 (OsPYL/RCAR3 overexpressing plant) and its control Dongjin were prepared with or without ABA treatment for 12 h. All total RNA were extracted from shoot and then purified using the RNeasy Mini Kit (Qiagen, Hilden, Germany). The quality control was conducted by Agilent Technologies 2100 Bioanalyzer (Agilent Technologies, Santa Clara, CA, USA). The libraries for sequencing were prepared using a TruSeqRNA Sample Prep Kit v2 (Illumina Inc., San Diego, CA, USA), following the manufacturer’s instructions. The sequencing of the libraries was performed using a HiSeq 4000 system (Illumina Inc., San Diego, CA, USA) generating single-end 101 bp reads. The trimmed reads were mapped to IRGSP (v1.0) and assembled into transcripts. The read counts were determined using the StringTig program, and then normalized into FPKM (fragments per kilobase of transcript per million mapped reads). Fold change (FC) was generated by log_2_[FPKM+1]. The graphs were constructed using *Prism6* (GraphPad Software, San Diego, CA, USA).

### 4.8. Protein Phosphatase Assay

Protein phosphatase activity assay was performed as described previously [49]. Briefly, 6xHis-OsPP2C50, 6xHis-OsPYL/RCAR3, 6xHis-OsPYL/RCAR10, and GST-OsPYL/RCAR7 were purified using *E. coli* recombinant protein overexpression system. Then, 80 nM of 6xHis-OsPP2C50 and 400 nM of OsPYL/RCAR protein each were added to reaction buffer (20 mM Tris-HCl pH 8.0, 1 mM MnCl_2_, 0.1% (*v/v*) 2-mercaptoethanol, and 0.1 mg/mL BSA) with various concentrations of (+)-ABA. Pre-incubation for 20 min at 37 °C was followed by adding 15 mM of pNPP (*ρ*-nitrophenyl phosphate) as a substrate for the protein phosphatase. The amount of *ρ*-nitrophenol was determined after 1 h incubation at 37 °C by measuring absorbance at 405 nm. IC_50_ values were calculated using *Prism 5* (GraphPad Software, San Diego, CA, USA) with the following equation: $y=\frac{100}{\left(1+10^{\left(x-\log IC50\right)}\right)}$, where *x* and *y* refer to relative phosphatase activity (%) and log scale of ABA concentration (μM), respectively.

### 4.9. Co-Immunoprecipitation Assay

For co-immunoprecipitation experiments, we used GFP-trap (ChromoTek, Martinsried, Germany) for the immunoprecipitation of green fluorescent protein (GFP)-fusion proteins. OsPYL/RCAR7 and OsPP2C51 were inserted into pENTR/D-topo vectors (Invitrogen, CA, USA) and then recombined with pGEM-gw-GFP vectors for GFP fusion, and pGEM-gw-3xHA vector for HA tagging using LR recombinase (Invitrogen, CA, USA) [21]. The indicated constructs were introduced into rice protoplasts using the polyethylene glycol (PEG)-mediated method, and the transformed protoplasts were incubated at 28 °C. Cellular extracts from transformed protoplasts in immunoprecipitation buffer (150 mM NaCl, 50 mM Tris-HCl at Ph 7.5, 1 mM EDTA, 2 mM EGTA, 2 mM MgCl_2_, 0.5% NP40, 0.5% Triton X-100, and 1× protease inhibitor cocktail (complete ULTRA tablet, Roche, IN, USA) were incubated with pre-cleaned GFP-trap beads at 4 °C for 2 h. After washing five times with immunoprecipitation buffer, the precipitated proteins, together with GFP-trap, were subjected to SDS-PAGE and immunoblot analysis. Precipitated GFP or HA tagged proteins were detected with anti-GFP rabbit antibody (Life Technologies, OR, USA) or anti-HA rat antibody (Roche, IN, USA), respectively.

### 4.10. Statistical Analysis

All statistical analyses were performed with the Graphpad Prism5 program. Variance was confirmed by one-way or two-way analysis of variance (ANOVA) test and Dunnett’s or Bonferroni multiple comparison, with significant *p*-value < 0.05. All samples were prepared with a minimum of three replicates.

## Supplementary Materials

Supplementary Materials can be found at https://www.mdpi.com/1422-0067/21/11/4163/s1.
