# Supplementary figures and images for: Ectopic Expression of OsPYL/RCAR7, an ABA Receptor Having Low Signaling Activity, Improves Drought Tolerance without Growth Defects in Rice
Source: Int J Mol Sci. 2020 Jun 11;21(11):4163. doi: 10.3390/ijms21114163 (PMC7312952; doi:10.3390/ijms21114163)

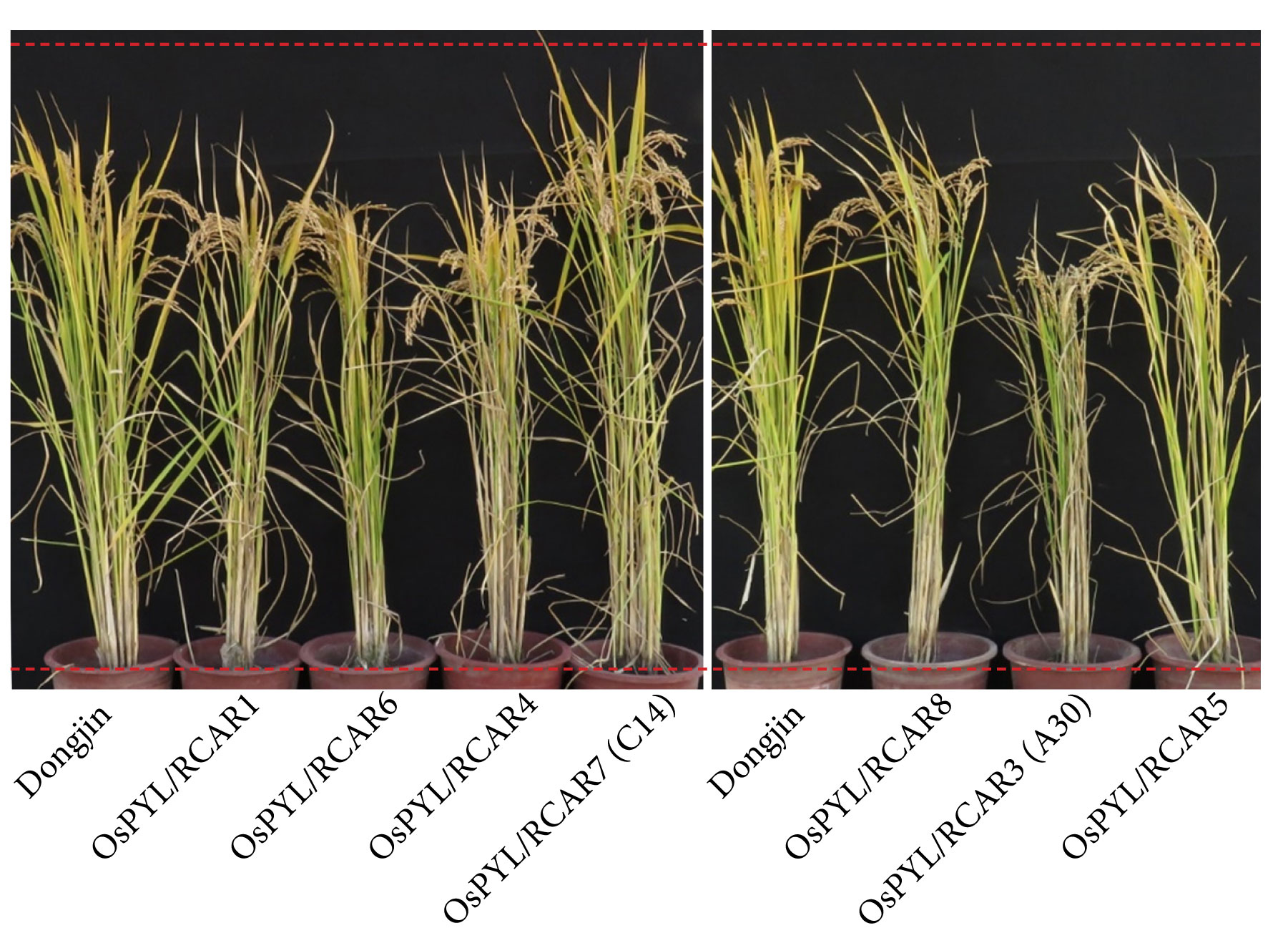

Supplement: Supplementary file 1 [file ijms-21-04163-s001.zip › Supplementary Figure 1.jpg]

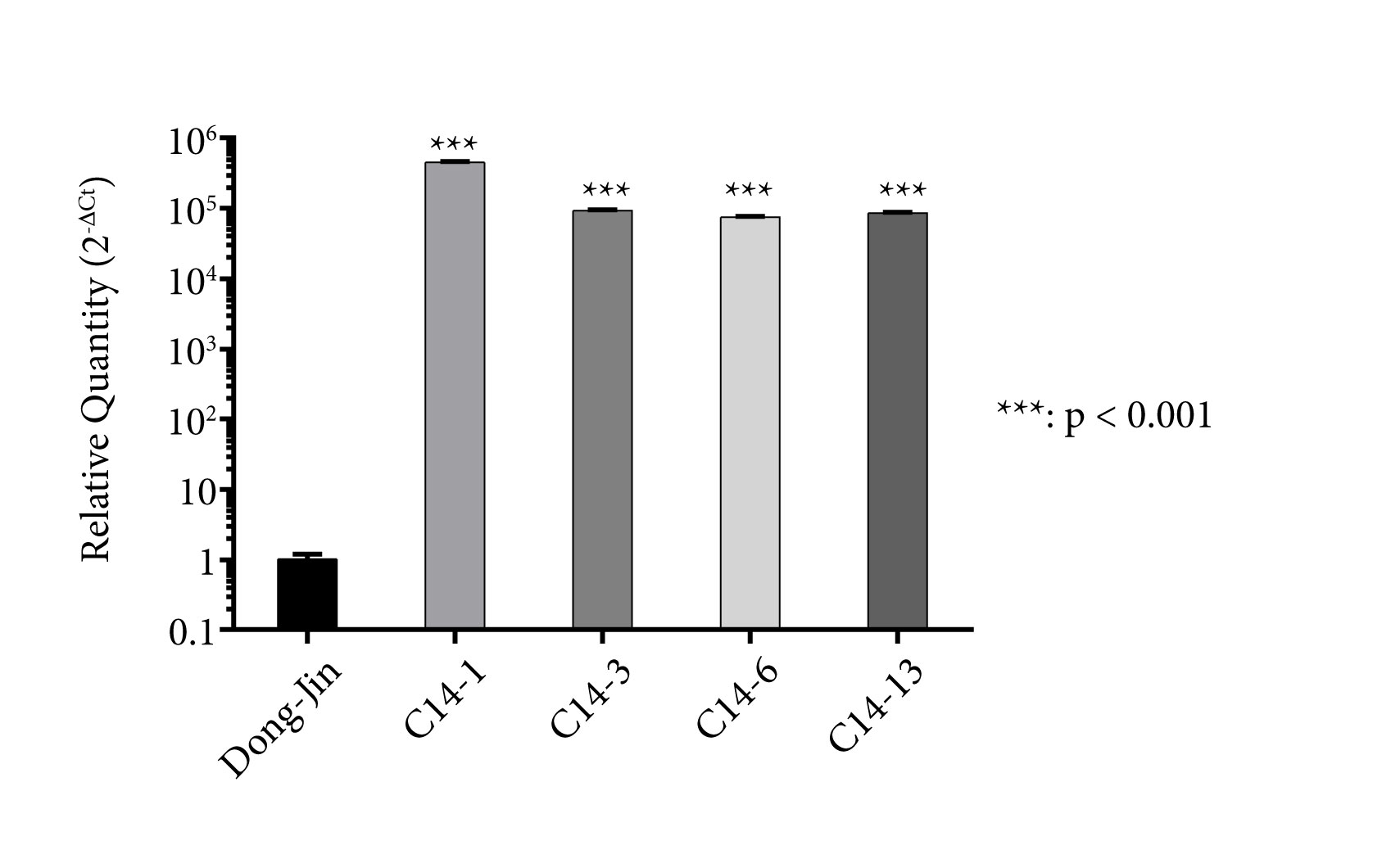

Supplement: Supplementary file 1 [file ijms-21-04163-s001.zip › Supplementary Figure 2.jpg]
